# Antithrombin supplementation during extracorporeal membrane oxygenation: study protocol for a pilot randomized clinical trial

**DOI:** 10.1186/s13063-019-3386-4

**Published:** 2019-06-11

**Authors:** Mauro Panigada, Elena Spinelli, Alberto Cucino, Elisa Cipriani, Stefano De Falco, Giovanna Panarello, Giovanna Occhipinti, Antonio Arcadipane, Gabriele Sales, Vito Fanelli, Luca Brazzi, Cristina Novembrino, Dario Consonni, Antonio Pesenti, Giacomo Grasselli

**Affiliations:** 1 Department of Anesthesia, Critical Care and Emergency, Fondazione IRCCS Ca’ Granda Ospedale Maggiore Policlinico, Milan, Italy; 2 Department of Pathophysiology and Transplantation, University of Milan, Milan, Italy; 3 Department of Anesthesiology and Intensive Care, ISMETT IRCCS (Istituto Mediterraneo per i Trapianti e Terapie ad Alta Specializzazione), UPMC, Palermo, Italy; 4 Department of Surgical Sciences, University of Turin, Turin, Italy; 5 Clinical Laboratory, Fondazione IRCCS Ca’ Granda Ospedale Maggiore Policlinico, Milan, Italy; 6 Epidemiology Unit, Fondazione IRCCS Ca’ Granda Ospedale Maggiore Policlinico, Milan, Italy

## Abstract

**Background:**

Normal levels of plasma antithrombin (AT) activity might decrease heparin requirements to achieve an adequate level of anticoagulation during treatment with extracorporeal membrane oxygenation (ECMO). Acquired AT deficiency during ECMO is common, but formal recommendations on target, timing, and rate of AT supplementation are lacking. Thus, we conceived a pilot trial to evaluate the feasibility and safety of prolonged AT supplementation in patients requiring veno-venous ECMO for respiratory failure.

**Methods:**

Grifols Antithrombin Research Awards (GATRA) is a prospective, randomized, single blinded, multicenter, controlled two-arm trial. Patients undergoing veno-venous ECMO will be randomized to either receive AT supplementation to maintain a functional AT level between 80 and 120% (AT supplementation group) or not (control group) for the entire ECMO course. In both study groups, anticoagulation will be provided with unfractionated heparin following a standardized protocol. The primary endpoint will be the dose of heparin required to maintain the ratio of activated partial thromboplastin time between 1.5 and 2. Secondary endpoints will be the adequacy of anticoagulation and the incidence of hemorrhagic and thrombotic complications.

**Discussion:**

GATRA is a pilot trial that will test the efficacy of a protocol of AT supplementation in decreasing the heparin dose and improving anticoagulation adequacy during ECMO. If positive, it might provide the basis for a future larger trial aimed at verifying the impact of AT supplementation on a composite outcome endpoint including hemorrhagic events, transfusion requirements, and mortality.

**Trial registration:**

ClinicalTrials.gov, NCT03208270. Registered on 5 July 2017.

**Electronic supplementary material:**

The online version of this article (10.1186/s13063-019-3386-4) contains supplementary material, which is available to authorized users.

## Background

Extracorporeal membrane oxygenation (ECMO) is a temporary life support method for patients with severe acute respiratory failure refractory to conventional treatment, and its use is continuously increasing worldwide [1]. Since exposure of blood to the non-biologic surface of the extracorporeal circuit induces a pro-thrombotic state and an inflammatory response, the use of ECMO necessitates the maintenance of hemostatic balance to minimize the risk of both hemorrhagic and thrombotic complications [2]. Consequently, to avoid clotting in the extracorporeal circuit and in the patient, anticoagulation is necessary, but it increases the risk of bleeding [3]. A recent retrospective analysis on more than 2000 patients reported bleeding and thrombotic complications with a frequency of up to 45% and 60%, respectively, with major impact on outcome [4].

Anticoagulation management during ECMO is usually based on continuous infusion of unfractionated heparin [5, 6]. The heparin effect is strictly dependent on antithrombin (AT) activity in plasma [7, 8]. Acquired AT deficiency during ECMO is common and multifactorial [9]: possible mechanisms include consumption due to activated coagulation and long-term anticoagulation, but also impaired synthesis, degradation by elastase from activated neutrophils, and disseminated intravascular coagulation. AT deficiency contributes to heparin resistance, with resulting difficulty in achieving therapeutic anticoagulation and increased heparin dose [7]. Theoretically, normalization of AT levels should decrease heparin requirements to achieve a proper anticoagulation target [9]. This may have a relevant clinical impact because risk of bleeding during ECMO is reasonably associated with higher heparin dosage, and a better control of anticoagulation may improve patients’ outcome [10]. However, formal recommendations on target, timing, and rate of AT supplementation during ECMO are lacking.

Given this lack of current knowledge, we designed a prospective randomized controlled clinical trial to evaluate the effects of a protocol of AT supplementation to achieve and maintain a normal AT activity on heparin dose, level of anticoagulation, bleeding, and thrombotic complications in adult patients undergoing ECMO for respiratory failure.

The results of this study will clarify some of the unanswered issues on AT supplementation during ECMO and will eventually provide the basis for a subsequent larger study on outcome.

## Methods

### Study design

**Fig. 1:**
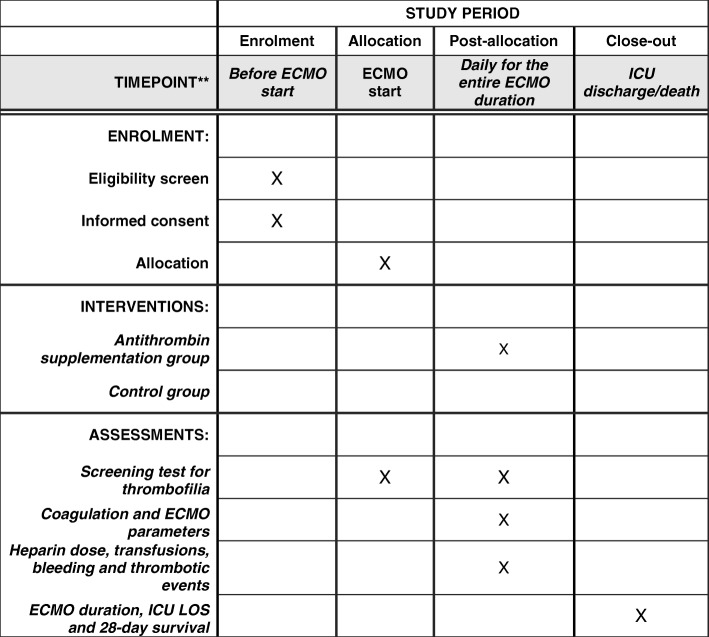
Intervention and assessment schedule

The Grifols Antithrombin Research Awards (GATRA) study is a pilot, prospective, randomized, single blinded, multicenter, controlled two-arm trial that will be performed on adult patients undergoing veno-venous ECMO for severe respiratory failure. The study will be conducted in adherence to the principles of the World Medical Association’s Declaration of Helsinki and in accordance with the Medical Research Involving Human Subjects Act (WMO). The Ethics Committee of the coordinating center (Comitato Etico Milano Area B) approved the protocol on April 18, 2017 (Version 1.6.7 - April 4, 2017), and approval of the local Ethics Committees will be required before starting the trial in the participating centers. The study was authorized by AIFA (Agenzia Italiana del Farmaco) on April 11, 2017 (EudraCT number 2016-004534-23) and registered at www.ClinicalTrials.gov with code NCT03208270. Informed consent to participate in the clinical trial will be obtained, and information on the clinical trial will be given to each patient. If the patient is not capable of giving informed consent at the time of enrollment, a deferred consent will be given. The informed consent will be sought by the investigator from the patient, and the information about the study will be given to the patient or his/her legally designated representative as soon as possible. If the patient does not consent to the study, he/she will be informed of the right to object to the use of data obtained from the clinical trial. The Standard Protocol Items: Recommendations for Interventional Trials (SPIRIT) checklist is provided in Additional file 1, and the SPIRIT figure is included in the main body of the manuscript (Fig. 1).

### Setting

The GATRA trial will be performed in the intensive care units (ICUs) of Italian ECMO referral centers. The coordinating center will be Fondazione IRCCS Ca′ Granda OspedaleMaggiore Policlinico in Milan, Italy.

### Study population

**Fig. 2:**
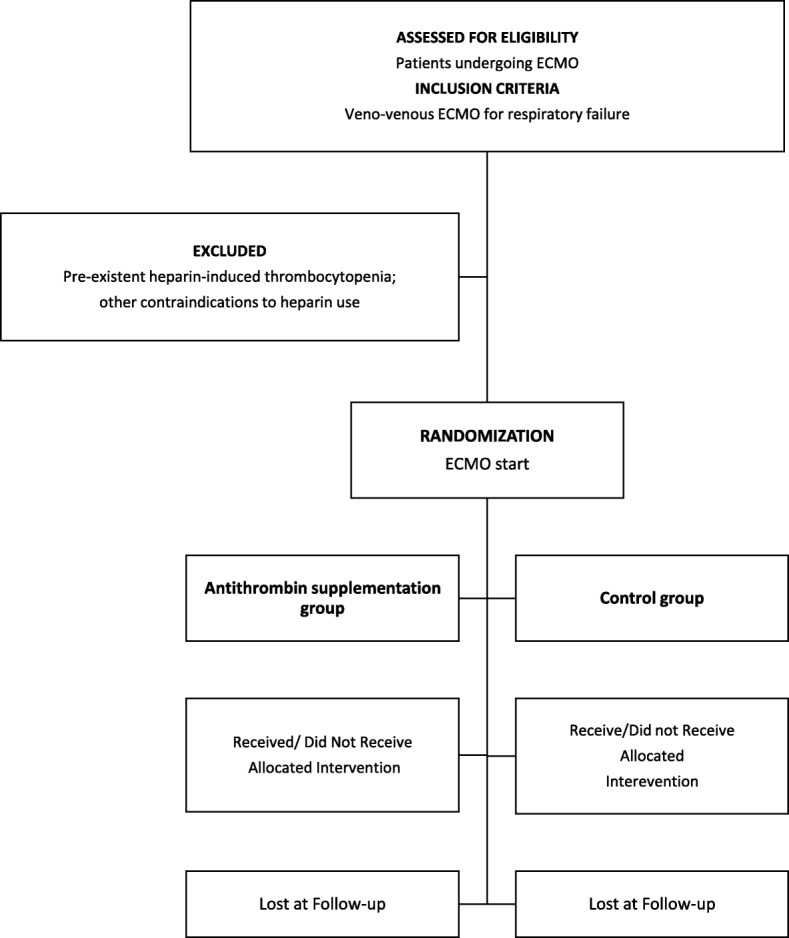
CONSORT flow diagram

Consecutive adult patients admitted to the participating centers requiring veno-venous ECMO as a support for respiratory failure will be enrolled in the study. Exclusion criteria will be pre-existing heparin-induced thrombocytopenia or other contraindications to heparin use (namely high risk of bleeding after major surgery prompting the decision to start ECMO without systemic anticoagulation). The Consolidated Standards of Reporting Trials (CONSORT) diagram of the GATRA trial is presented in Fig. 2.

### Randomization

As soon as the clinical decision to initiate ECMO is made, the patient will be enrolled in the study. Patients will be block randomized per center through an online automatic centralized and computerized system to one of the two groups (1:1 ratio): AT supplementation group or control group.

### Interventions

#### Antithrombin supplementation group

**Fig. 3:**
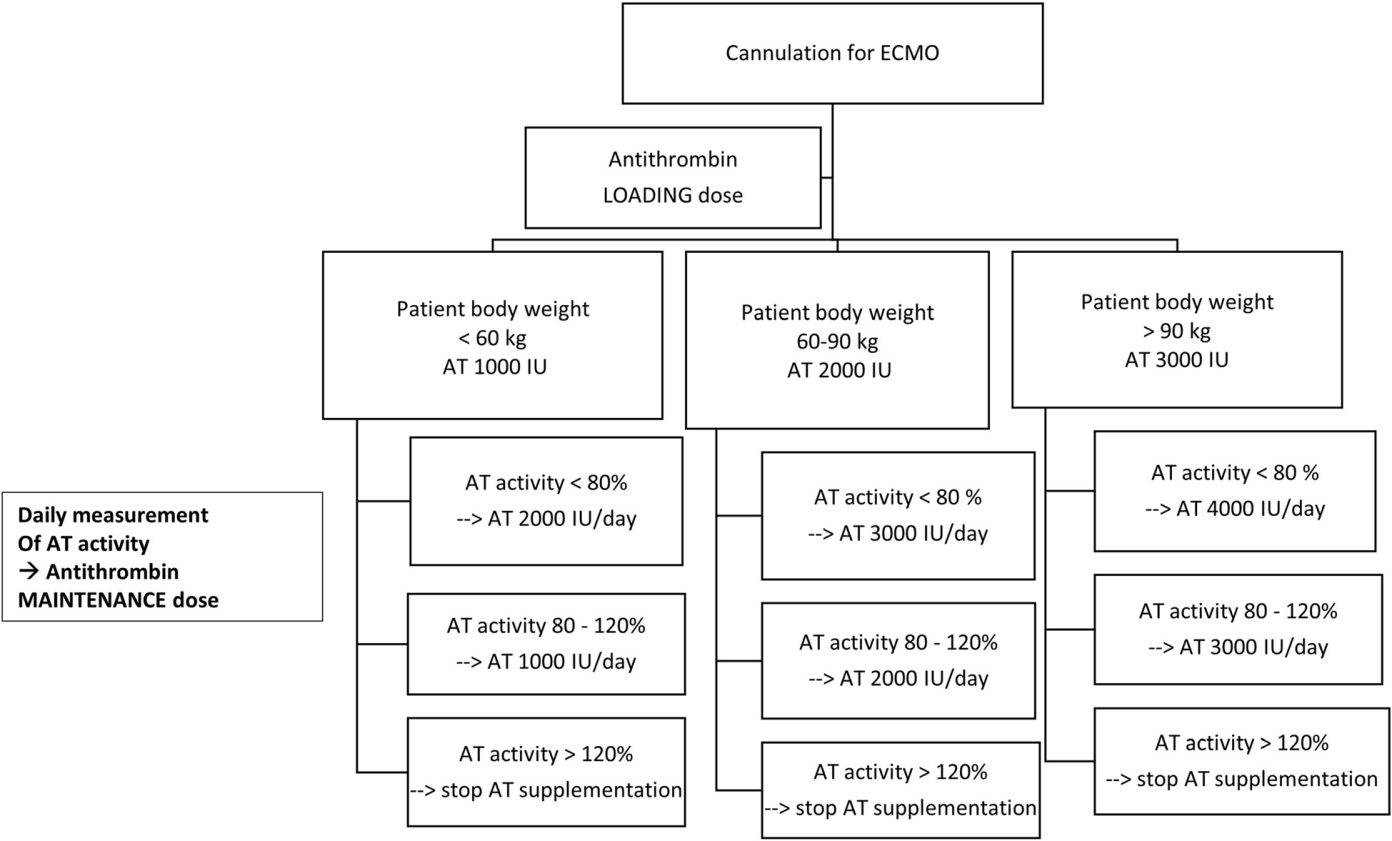
Protocol for anthithrombin supplementation

Patients randomized to the AT supplementation group will receive supplementation of AT concentrate to maintain a functional AT level between 80 and 120%. The AT level will be measured prior to ECMO start and then once daily and supplementation adjusted accordingly. In the study group, AT concentrate supplementation will be interrupted when AT levels exceeds 120% and resumed as soon as the levels drop below 80%. The protocol for AT supplementation is illustrated in Fig. 3.

#### Control group

Patients randomized to the control group will not receive supplementation of AT unless heparin resistance occurs. The following safety criteria will be applied to define “heparin resistance”: when a patient in the control group requires more than 35 IU/kg/h of heparin and his/her AT plasma level is equal to or lower than 50%. Dosage regimen for AT supplementation in the control group will follow the same supplementation algorithm as in the treatment group. AT will be supplemented to achieve an AT plasma level higher than 50%. The AT level will be measured prior to ECMO start and then once daily.

#### Anticoagulation protocol for both groups

**Fig. 4:**
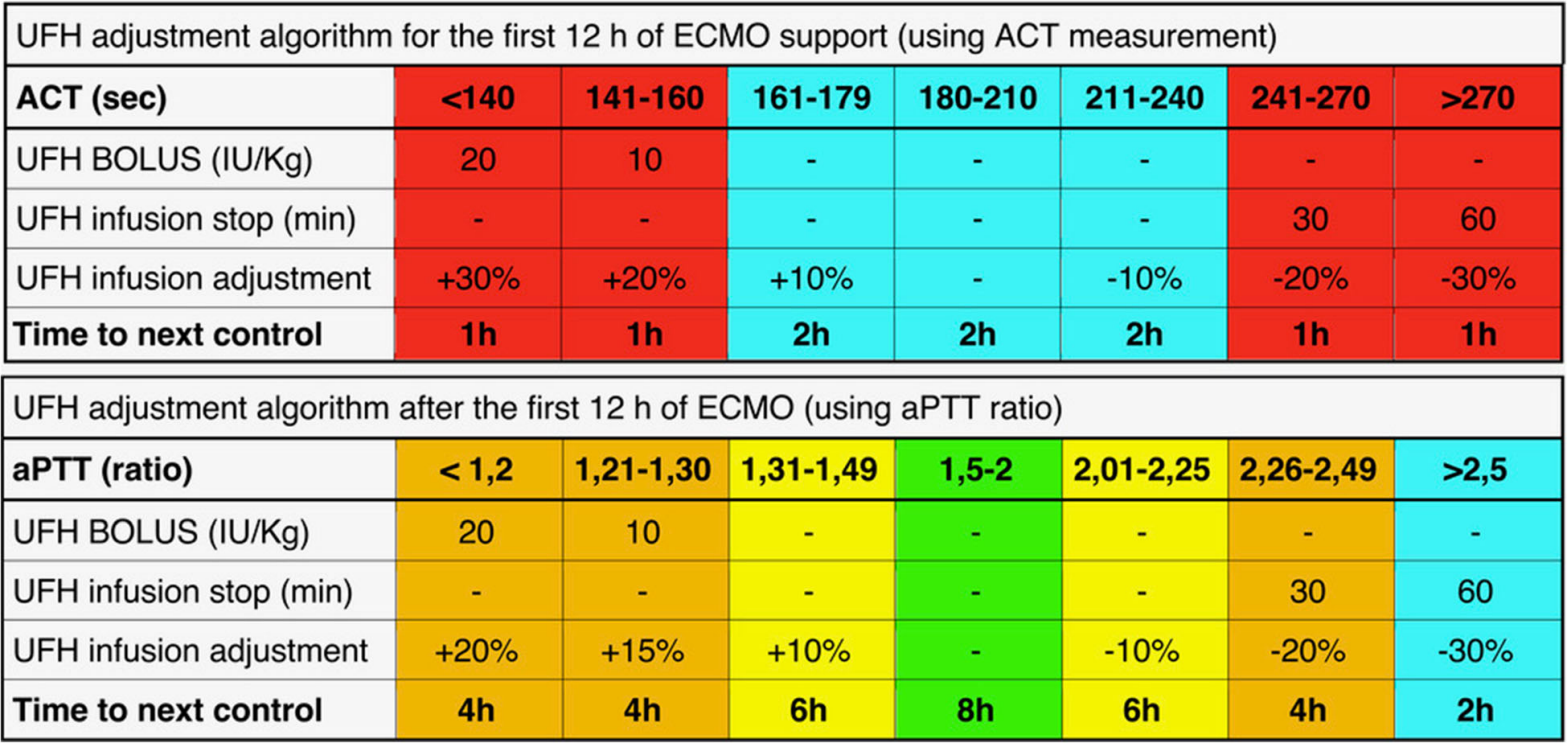
Anticoagulation protocol

In both study groups, anticoagulation during ECMO will be provided with unfractionated heparin following the institutional protocol of the coordinating center, as previously published [10] (Fig. 4). Briefly, a heparin bolus of 50–70 IU/kg, depending on baseline activated partial thromboplastin time (aPTT) value, will be administered at ECMO start, followed by 18 IU/kg/h continuous infusion. For the first 12 h after ECMO start, anticoagulation will be monitored with activated clotting time (ACT) (therapeutic range 180–210 s, performed every 1 or 2 h according to the standardized protocol). From the 13th hour on, anticoagulation will be guided by aPTT, with a target aPTT ratio (patient-to-normal) range of 1.5–2.0. The frequency of aPTT measurements may vary from a minimum of three times per day to a maximum of six depending on the standardized protocol. When the aPTT value falls well below the desired range, a bolus of heparin will be administered. When the value is too high, infusion will be stopped for either 30 or 60 min. In both groups the following safety criteria will be used: in case of major surgery, during the first 24 h after operation, minimal levels of anticoagulation will be allowed to minimize the risk of bleeding. In case of minor bleeding (i.e., dripping from the site of the vascular catheter’s cannulation), physicians shall maintain anticoagulation targeted to the lower level (i.e., aPTT ratio 1.5). In case of clinically significant bleeding, heparin infusion may be interrupted until bleeding ceases; infusion will be restored starting at 50–70% of the pre-existing dose. No changes will be made to the AT dosage regimen.

#### Criteria for transfusions of blood products and replacement of the ECMO circuit

For the entire duration of the study, hemoglobin will be maintained at > 10 g/dL, platelet count > 50,000/mm^3^, and fibrinogen > 150 mg/dL, through packed red blood cells, platelets, and fresh frozen plasma and/or fibrinogen concentrate transfusions, respectively.

To standardize the clinical decision to replace the ECMO circuit with a new one, an ad hoc “circuit change-out score” was developed that considers the performance of the oxygenator, signs of coagulation activation by the extracorporeal circuit (e.g., drop in platelet count, increase in D-dimer level, and decrease in fibrinogen level), and the presence of hemolysis. The “circuit change-out score” includes two different conditions (A and B) defined by different sets of criteria. Condition A requires all the following three criteria to be satisfied: decrease in platelet count greater than 50% in 48 h; decrease in fibrinogen level greater than 33% in 48 h; increase in D-dimer level greater than 100% with D-dimer level higher than 30,000 ng/mL. Condition B requires two of the following three criteria to be satisfied: platelet count lower than 50,000/mL; fibrinogen lower than 150 mg/dL; D-dimer higher than 50,000 ng/mL, provided that D-dimer elevation is one of the two “B” conditions.

#### Stopping rules

Patients will be supplemented with AT during ECMO according to the assigned group and followed up for the entire duration of ECMO. AT supplementation will be stopped at ECMO discontinuation.

Premature discontinuation of the study will be allowed when one of the following conditions occurs: (1) death; (2) onset of heparin-induced thrombocytopenia requiring shift to an alternative anticoagulant drug to heparin; (3) unexpected circumstances that prevent proper heparin or AT supplementation.

#### End of follow-up

Enrolled patients will be observed until ICU discharge or death, whichever comes first.

### Study endpoints

#### Primary endpoint

The primary endpoint will be the amount of heparin infused to each patient, calculated as the total administered heparin per kilogram of body weight (including boluses) divided by the total duration of heparin infusion in hours (IU/kg/h).

#### Secondary endpoints

Adequacy of anticoagulation will be assessed through anti-factor Xa levels.

The study will also provide a preliminary evaluation of the safety of AT supplementation by comparing the incidence of the following adverse events in the two study groups: bleeding complications, classified according to a modified version of the Bleeding Academic Research Consortium score to our clinical settings [11, 12]; blood products transfusion requirements; patient’s or circuit thrombosis.

### Data collection

At enrollment, we will anonymously collect patients’ demographic information (e.g., age, sex, height, weight), past (e.g., chronic medications including anticoagulant and antiplatelet drugs) and recent (e.g., etiology of the acute respiratory failure) medical history, and ECMO configuration with cannulation sites. A blood sample will be drawn from patients to test inherited or acquired thrombophilia (proteins C and S, Factor (F) V *G1691A* and FII *G20210A*, lupus anticoagulant, anti-cardiolipin, and anti-β2-glycoprotein 1 antibodies).

The following data will be recorded every day for the whole study duration: daily heparin dosage; coagulation parameters (including D-dimer, fibrinogen, aPTT ratio, prothrombin time (PT) ratio, anti-factor Xa, antithrombin activity, ACT); complete blood count; indexes of hemolysis (hemoglobin, free hemoglobin, and haptoglobin levels); transfusion of blood products.

Bleeding and thrombotic events will also be recorded. Within 24 h after ECMO removal, a Doppler ultrasonography of the cannulated vessels and of the vena cava will be performed to exclude thrombosis due to vascular cannulation. Functional ECMO parameters will be evaluated once per day (membrane oxygenator shunt, blood flow resistance, oxygenation performance) and recorded.

Evaluation of clinical outcome will include duration of ECMO, ICU length of stay, and survival at 28 days.

Collected data will be entered in an electronic case report form (eCRF) available online at a dedicated website (https://ecmostudy.fbk.eu), with protected individual access for each participating center. Patient data will be anonymous and coded according to a number. The eCRF includes tools to promote data quality, such as range checks for data values. Data monitoring will be performed by means of queries on the database done by statisticians and analyzed to identify abnormalities and inconsistencies.

### Statistical considerations

All statistical analyses will be done with Stata version 14.1 (StataCorp, College Station, TX, USA) in the coordinating centers at the Epidemiology Unit of Fondazione IRCCS Ca′ Granda Ospedale Maggiore Policlinico in Milan, Italy.

#### Sample size

Sample size is calculated using the confidence interval approach upon the primary outcome measure of reduction of heparin dose in the study group treated with AT to maintain a normal AT plasma level (considered to be a level higher than 80%) compared to the control group. Preliminary data from patients in ECMO, cared for with the same heparin titration protocol of this study, showed that the mean heparin dosage was 18 IU/kg/h and 13 IU/kg/h in patients with AT activity lower and higher than the median (73.5%), respectively. Assuming an estimated difference of 5 IU/kg/h of heparin between the two groups, a sample size of 40 patients (20 in each treatment group) would yield a 95% confidence interval of average heparin reduction from 1 to 9 IU/kg/h).

#### Proposal for analysis

Baseline variables will be compared between each randomized arm to assess the randomization performance and baseline balance between arms. We will not perform statistical testing for baseline comparison between arms, because any differences in baseline characteristics are consequences of chance due to randomization. All analyses will be conducted in accordance with the intention-to-treat principle and in accordance with the CONSORT statement.

For the primary and secondary outcomes, continuous variables will be reported as means ± standard deviations or medians and 25–75% interquartile ranges. Categorical variables will be reported as absolute and relative frequencies. Differences between groups will be tested using Mann-Whitney and chi-square or Fisher’s exact tests, as appropriate. Estimates of the effects and their confidence intervals will also be reported. In case of evident imbalances for key baseline variables between treated patients and controls, we will perform statistical adjustment using regression models.

For repeated outcomes within subject, the association between AT dosage, heparin dosage, bleeding, and monitoring methods will be evaluated using random-effect models.

### Study organization

This is an investigator-initiated trial. Two principal investigators (MP and GG) designed the study protocol. Each participating center will indicate a local investigator in charge of the study. The principal investigators are responsible for administrative management and communication with the local investigators and for helping the participating clinical sites in trial management, record keeping, and data management. The study will be conducted with the financial support of Grifols (GATRA – Antithrombin Research Awards 2016). Funders will not have any role in the analysis and interpretation of the study results or in the decision to submit the report for publication. The local investigators guarantee the integrity of data collection. All adverse events will be monitored by the coordinating center and reported to AIFA according to the national legislation. Specific patient insurance will be granted to cover all unexpected adverse events caused by the study interventions.

### Ancillary study

The rationale for AT supplementation during ECMO is also based on its coagulation-independent anti-inflammatory effects. Some of these mechanisms include endothelial release of prostacyclin that inhibits aggregation and activation of platelets [13, 14] and the reduction of various cytokines and chemokines from the endothelial cells [15, 16].

To evaluate markers of inflammation and epithelial and endothelial damage, 4 aliquots of plasma, 0.5 mL each, will be obtained from each patient in the GATRA study, by centrifugation of ethylenediaminetetraacetic acid (EDTA) blood at 3000 revolutions per minute (RPM) for 15 min at 4 °C, prior to ECMO start, 24 h after ECMO start, 72 h after ECMO start, before ECMO removal, and 7 days after ECMO removal (or before discharge from the ICU if this happens first). Then the aliquots will be stored, shortly after collection, at − 80 °C until levels of tumor necrosis factor (TNF)-α, interleukin (IL)-1β, IL-6, IL-8, IL-10, endocan, and syndecan are measured. At the same time points we will also collect blood to evaluate the transcriptional profile of circulating cells in whole blood. To identify changes in the transcriptional activation of genes involved in inflammation, epithelial, and endothelial damage, 2.5 mL of whole blood will be collected in RNA PAXgene tubes prior to ECMO start and 24 and 72 h after ECMO start. Specimens will be stored at − 20 °C until transcriptome profiling is performed [17].

## Discussion

GATRA is a pilot randomized controlled trial aimed at testing a protocol of AT supplementation in patients on ECMO for severe respiratory failure. It will assess the feasibility and the safety of prolonged AT supplementation to target normal AT levels and its efficacy in decreasing the heparin dose and improving anticoagulation adequacy. If positive, it might provide the basis for a future larger trial aimed at verifying the impact of AT supplementation on a composite outcome endpoint including hemorrhagic events, transfusion requirements, and mortality.

### Antithrombin supplementation during ECMO

Heparin mainly exerts its anticoagulant effect by binding AT and facilitating the inhibition of thrombin by AT. When heparin binds to AT, it converts AT from a slow to a rapid thrombin inhibitor. Therefore, the therapeutic activity of heparin depends on the availability of circulating AT. The heparin/AT also inhibits factor Xa and to a lesser extent other activated coagulation factors (Xa, IXa, XIa, and XIIa) [18]. Another possible anticoagulant mechanism of heparin includes the release of tissue factor pathway inhibitor from endothelial cells [19]. Although AT is needed by heparin to properly exploit its anticoagulant activity, there is no consensus on AT supplementation during ECMO. Guidelines suggest supplementing AT in ECMO only when its deficiency coexists with heparin resistance [6]. Few studies evaluated the effect of AT supplementation during ECMO without a consensus on the target level to be maintained. Small and retrospective studies in the pediatric population examined the administration of AT during ECMO but yielded inconclusive results, with some reporting no effects on heparin dose and bleeding [20–22] and others observing decreased heparin requirements [23–25] and fewer transfusions [25]. These contrasting results might be explained by the high variability in dosing schemes, target levels, timing, and duration of AT supplementation.

### Development of the protocol for AT supplementation: practical and operational issues

Since dosing recommendations are not provided for acquired AT deficiency, the protocol for AT supplementation was developed following manufacturer recommendations for hereditary deficiency. Supplementation dosing is based on patient body weight and the difference between the actual and the desired AT activity. A level of AT activity corresponding to the normal range (80–120%) was chosen as the target for supplementation. The calculated dose was rounded up to the nearest vial size, and dose tiers were created using ranges of AT activity to minimize bedside calculations. The protocol mandates, for patients in the AT supplementation group, the administration of a loading dose of AT upon ECMO cannulation. As the level of AT activity before ECMO start was expected not to be available for most patients, the loading dose is based solely on the patient body weight. Subsequent dose adjustments are based on the patient body weight and the plasma AT activity, which must be measured daily. Patients with a plasma AT activity between 80 and 120% will receive a maintenance dose, which will be increased by 1000 IU if the plasma AT activity is lower than 80%. Observational studies suggest that intermittent administration of AT is less effective than continuous infusion in correcting AT deficiency; moreover, rapid AT binding to heparin complexes after bolus administration could theoretically increase the risk for bleeding. Based on the clinical experience of our and other ECMO centers [26, 27], administration of AT via an extended infusion decreases heparin rate modifications and avoids AT levels fluctuations, without increasing the risk of major bleeding. Therefore, the protocol prescribes AT administration by extended infusion both for loading and maintenance doses.

### Study endpoints

Hemorrhagic complications remain a major cause of morbidity and mortality in ECMO patients [4]. Hence, protocols to guide anticoagulation management are key in the management of these patients. Physiological considerations suggest that normalization of AT levels might improve adequacy of heparin anticoagulation and possibly decrease the risk of bleeding. Indeed, in adult patients undergoing ECMO for respiratory failure, lower AT levels were associated with increased need for transfusion [26], and AT supplementation facilitated achievement of target anticoagulation without increasing heparin dosage [27]. A higher degree of anticoagulation [28] is associated with hemorrhagic events during ECMO, but risk of bleeding is multifactorial and does not depend solely on anticoagulation. We chose the reduction of heparin dose as the primary endpoint for this pilot trial; however, it is conceivable that larger studies will be needed to detect the hypothesized effect on clinical outcome.

### Study strengths and limitations

One of the strengths of the study is that the protocol contains a well-defined heparin anticoagulation protocol, which has already been adopted in a previous multicenter trial [29]. This should facilitate its application and minimize deviations in anticoagulation management which could jeopardize the effect of AT supplementation on study endpoints. Although the decision to transfuse blood products is clinical, the protocol recommends well-defined targets for hemoglobin, platelet count, and fibrinogen. Similarly, criteria for considering replacement of the ECMO circuit are provided.

One limitation of the GATRA trial is that blinding is not possible due to the nature of the intervention. However, the factors that can potentially affect the primary outcome (in particular, heparin dose adjustments and transfusion of blood products) are regulated by the protocol.

### Trial status

The GATRA trial is currently recruiting patients. The protocol was approved by the Ethics Committee of the coordinating center on April 18, 2017. The first patient was enrolled on August 8, 2017. As of September 9 2018, 36 patients have been screened and 23 patients have been randomized (11 interventional group, 12 control group) in the coordinating center. Recruitment is expected to be completed in May 2019.

## Additional file

Additional file 1: SPIRIT 2013 checklist. (DOC 121 kb)

## Abbreviations

ACT: Activated clotting time
AIFA: Agenzia Italiana del Farmaco
aPTT: Activated partial thromboplastin time
AT: Antithrombin
ECMO: Extracorporeal membrane oxygenation
eCRF: Electronic case report form
EDTA: Ethylenediaminetetraacetic acid
ICI: Intensive care unit
IL: Interleukin
IU: International Unit
OR: Odds ratio
PT: Prothrombin time
RPM: Revolutions per minute
TNF: Tumor necrosis factor
